# Significantly elevated number of human infections with H7N9 virus in Jiangsu in eastern China, October 2016 to January 2017

**DOI:** 10.2807/1560-7917.ES.2017.22.13.30496

**Published:** 2017-03-30

**Authors:** Xiang Huo, Liling Chen, Xian Qi, Haodi Huang, Qigang Dai, Huiyan Yu, Yu Xia, Wendong Liu, Ke Xu, Wang Ma, Jun Zhang, Changjun Bao

**Affiliations:** 1 Jiangsu Provincial Center for Disease Control and Prevention, Nanjing, China; 2 These authors contributed equally to this work; 3 Suzhou Center for Disease Control and Prevention, Suzhou, China; 4 Nanjing Medical University, Nanjing, China

**Keywords:** Human, H7N9, influenza, mutation, pandemic, meteorology

## Abstract

Since first identified in 2013, the H7N9 virus has caused several waves of human infections in China, with a current wave including a number of patients with very severe disease. Jiangsu is one of the most impacted provinces, whereby as of 31 January 2017, the number of human infections (n = 109) in the ongoing fifth H7N9 wave has exceeded the sum of those in the four preceding ones. Ten of 13 cities in Jiangsu have been affected, and clustered infections as well as one co-infection with seasonal influenza have been observed. With a median age of 58 years and 74.3% (81/109) of patients being male, the characteristics of cases are similar to those in previous waves, however patients with H7N9 seem to have an accelerated disease progression. Preliminary case fatality remains above 30%. No significant viral mutations have been found in key functional loci. Environmental H7N9 detection rate and number of days with high risk ambient temperatures were both significantly elevated during the month of December 2016 when most human infections were reported. A number of municipal governments in Jiangsu have implemented live poultry market closures to impede viral transmission to humans. A detectable decline in human infections has been observed in these municipalities and the entire province since January 2017.

## Introduction

Avian influenza viruses cause human infections continuously worldwide. Compared with other viruses, such as H5N1, H5N6, H9N2 and H7N7, the H7N9 virus is much more potent due to its better ability to cross the species barriers and infect both poultry and humans, and this has raised serious concerns for potential pandemics [1]. As of 16 January 2017, a total of 918 laboratory-confirmed cases of human infection with H7N9 virus in China have been reported to the World Health Organization (WHO) in less than four years (since March 2013) [2]. In contrast, at the same date, and for a period of over 13 years (since 2003) the number of H5N1 cases worldwide was only 856 [2].

Since its first identification in March 2013, the H7N9 virus has caused five waves of human infections in China [3]. There were 134 cases, 304 cases, 219 cases and 118 cases detected and reported in the first four waves, respectively, with a declining trend in incidence [4]. However, the fifth wave (since September 2016) surged with a steep increase in case numbers from 1 December 2016, and 106 cases were reported in December 2016 alone. As of 31 December 2016, the number of reported cases in the fifth wave was 11.4, 2.7 and 6.1 times that of the corresponding periods in the second (10 cases), third (31cases) and fourth (16 cases) waves, respectively. Seven provinces in China have been affected, with Jiangsu being one of the most impacted [3]. Overall, the majority of reported human infections occurred in older males (median age: 57 years). The general case fatality was around 41% [4]. Here we describe the current fifth wave of human infections with H7N9 in Jiangsu province, which was characterised by a significantly elevated incidence in a wider affected area. Factors potentially contributing to the epidemic, such as meteorological factors, environmental detection rates of H7N9, and viral mutations, are also explored and discussed.

## Methods

### Human surveillance

In China, all laboratory-confirmed human infections with H7N9 are reported through a national system for reporting of notifiable infectious diseases [5]. In Jiangsu province, respiratory samples from suspected H7N9 patients are tested for H7N9 virus as well as common types of seasonal influenza (such as H1N1, H3N2 and B) by local municipal Centers for Disease Control and Prevention (CDC) using real-time PCR. The H7N9 avian influenza nucleotide test kits (bioPerfectus technologies, Taizhou, China) are most commonly used. The demographic, epidemiological and clinical information of patients infected with H7N9 is collected using standardised questionnaires by local Centers for Disease Control and Prevention (CDC) staff, or trained clinical doctors, and reported to Jiangsu Provincial CDC and China CDC through this system. Jiangsu Provincial CDC is responsible for checking and monitoring the reported information and takes part in the patients’ investigations if necessary. According to the Diagnosis and Treatment Scheme published by the National Health and Family planning commission of China, patients with pneumonia and either respiratory failure or any other organ dysfunction are considered as severe infections. 

All patients infected with H7N9 as of 31 January 2017 in Jiangsu province (n = 213) were included in this study. All the positive samples (confirmed by real-time PCR) collected from H7N9 patients were sent by local municipal CDCs to Jiangsu Provincial CDC, where the viruses were isolated and sequenced according to a previously described procedure [6].

### Environmental surveillance

Aiming to predict and assess risk of human infections, a surveillance on avian influenza virus in avian-associated sites, such as poultry farms, live poultry whole sale and retail markets, has been routinely conducted year-round in Jiangsu province since October 2013. The surveillance sites cover all 13 cities of Jiangsu province. An average of three to eight sites are covered monthly in each city, where swab samples of avian faeces, cages, or drinking water of birds and poultry for sale, are collected by each municipal CDC. All the samples are tested for avian influenza using real-time PCR by municipal CDCs and then results are reported to Jiangsu Provincial CDC.

### Meteorological factors

Air temperature has been previously reported to be associated with human infection with H7N9 virus, and higher risk of human infection was found when daily minimum temperatures range from ca 5 to 9 °C and when daily maximum temperatures are between ca 13 to 18 °C [7]. To investigate if air temperatures in Jiangsu might have been favourable to human infections during the fifth H7N9 wave, daily meteorological data provided by Jiangsu Provincial Meteorological Service Center were investigated.

### Phylogenetic analysis

The haemagglutinin (HA) nt sequences were edited and assembled using SeqManPro (DNASTAR,Madison,WI). ClustalXv.2.1 was used for the alignment of nt sequences [8]. A phylogenetic tree of the HA1 coding nt sequences was generated by Molecular Evolutionary Genetic Analysis (MEGA) version 6.06 [9] using a neighbour-joining method with 1,000 bootstrap replicates. We acknowledge the authors, originating and submitting laboratories of the sequences from the EpiFlu Database of the Global Initiative on Sharing Avian Influenza Data (GISAID) (Table 1).

**Table 1:** Origin of the haemagglutinin sequences of influenza A(H7N9) used for the phylogenetic analysis in this study

Segment ID	Segment	Country	Collection date	Isolate name	Originating Laboratory	Submitting Laboratory	Authors
EPI872958	HA	Hong Kong SAR	2016-Dec-28	A/Hong Kong/VB16189623/2016	Public Health Laboratory Services Branch, Centre for Health Protection	Public Health Laboratory Services Branch, Centre for Health Protection	Mak,G.C.; Lo,J.Y.C.
EPI887844	HA	China	2016-Dec-09	A/Guangdong/60060/2016	Guandong Centers for Disease Control	WHO Chinese National Influenza Center	Wang,Dayan;Zhou,Shumei;Li,Xiyan;Li,Xiaodan;Zhang,Ye;Bo,Hong;Shu,Yuelong
EPI887836	HA	China	2016-Dec-28	A/Guangdong/60923/2016	Guandong Centers for Disease Control	WHO Chinese National Influenza Center	Wang,Dayan;Zhou,Shumei;Li,Xiyan;Li,Xiaodan;Zhang,Ye;Bo,Hong;Shu,Yuelong
EPI887772	HA	China	2016-Dec-21	A/Zhejiang/11/2016	Zhejiang Provincial Center for Disease Control and Prevention	WHO Chinese National Influenza Center	Wang,Dayan;Zhou,Shumei;Li,Xiyan;Li,Xiaodan;Zhang,Ye;Bo,Hong;Shu,Yuelong
EPI887748	HA	China	2016-Dec-23	A/Zhejiang/14/2016	Zhejiang Provincial Center for Disease Control and Prevention	WHO Chinese National Influenza Center	Wang,Dayan;Zhou,Shumei;Li,Xiyan;Li,Xiaodan;Zhang,Ye;Bo,Hong;Shu,Yuelong
EPI887692	HA	China	2016-Dec-27	A/Zhejiang/18/2016	Zhejiang Provincial Center for Disease Control and Prevention	WHO Chinese National Influenza Center	Wang,Dayan;Zhou,Shumei;Li,Xiyan;Li,Xiaodan;Zhang,Ye;Bo,Hong;Shu,Yuelong
EPI887668	HA	China	2016-Dec-30	A/Zhejiang/20/2016	Zhejiang Provincial Center for Disease Control and Prevention	WHO Chinese National Influenza Center	Wang,Dayan;Zhou,Shumei;Li,Xiyan;Li,Xiaodan;Zhang,Ye;Bo,Hong;Shu,Yuelong
EPI887652	HA	China	2017-Jan-05	A/Zhejiang/6/2017	Zhejiang Provincial Center for Disease Control and Prevention	WHO Chinese National Influenza Center	Wang,Dayan;Zhou,Shumei;Li,Xiyan;Li,Xiaodan;Zhang,Ye;Bo,Hong;Shu,Yuelong
EPI888052	HA	China	2016-Dec-19	A/Anhui/60934/2016	Anhui Provincial Center for Disease Control and Prevention	WHO Chinese National Influenza Center	Wang,Dayan;Zhou,Shumei;Li,Xiyan;Li,Xiaodan;Zhang,Ye;Bo,Hong;Shu,Yuelong
EPI888036	HA	China	2016-Dec-13	A/Anhui/60931/2016	Anhui Provincial Center for Disease Control and Prevention	WHO Chinese National Influenza Center	Wang,Dayan;Zhou,Shumei;Li,Xiyan;Li,Xiaodan;Zhang,Ye;Bo,Hong;Shu,Yuelong
EPI887996	HA	China	2016-Dec-22	A/Anhui/60924/2016	Anhui Provincial Center for Disease Control and Prevention	WHO Chinese National Influenza Center	Wang,Dayan;Zhou,Shumei;Li,Xiyan;Li,Xiaodan;Zhang,Ye;Bo,Hong;Shu,Yuelong
EPI887852	HA	China	2016-Nov-20	A/Fujian/56600/2016	Fujian Provincial Center for Disease Control and Prevention	WHO Chinese National Influenza Center	Wang,Dayan;Zhou,Shumei;Li,Xiyan;Li,Xiaodan;Zhang,Ye;Bo,Hong;Shu,Yuelong
EPI887620	HA	China	2017-Jan-06	A/Fujian/02152/2017	Fujian Provincial Center for Disease Control and Prevention	WHO Chinese National Influenza Center	Wang,Dayan;Zhou,Shumei;Li,Xiyan;Li,Xiaodan;Zhang,Ye;Bo,Hong;Shu,Yuelong
EPI887612	HA	China	2017-Jan-01	A/Fujian/02151/2017	Fujian Provincial Center For Disease Control and Prevention	WHO Chinese National Influenza Center	Wang,Dayan;Zhou,Shumei;Li,Xiyan;Li,Xiaodan;Zhang,Ye;Bo,Hong;Shu,Yuelong

HA: haemagglutinin; WHO: World Health Organization.

### Ethical statement

The National Health and Family Planning Commission decided that the collection of data from cases of H7N9 was part of the public health investigation of the emerging outbreak, and thus the investigation was exempt from institutional review board assessment [10]. The dataset was anonymised in the national reporting system except for individuals with special access, and was anonymised before data analyses.

### Statistical analysis

Median and interquartile ranges (IQRs) were calculated for continuous variables and absolute numbers and proportions for categorical variables. Selected demographic, epidemiological and clinical characteristics of H7N9 patients were compared among five epidemic waves (March to April in 2013, December 2013 to May 2014, October 2014 to May 2015, December 2015 to May 2016 and October 2016 to 31 January 2017). Pearson chi-squared test was used for comparing proportions and continuity correction or Fisher’s exact test was used if appropriate. Kruskal–Wallis H test was used for comparing medians among multiple groups. Statistical analyses were conducted using R version 3.0.2 and statistical significance level was set at ≤ 0.05. The hierarchical colour map was produced with ArcGIS software version 10.0 (ESRI, Redlands, CA, US) to state the spatial patterns of human infections with H7N9. All cases with missing data on a certain characteristic were excluded when this characteristic was analysed. Information of total and missing data for each studied variable is shown in detail in Table 2. 

**Table 2:** Number of missing/total data of selected characteristics of H7N9 patients in Jiangsu province, March 2013–January 2017

Selected characteristics	Proportion of patients among the total missing information on a characteristic				
	Wave 1 (Mar to Apr 2013)	Wave 2 (Dec 2013 to May 2014)	Wave 3 (Oct 2014 to May 2015)	Wave 4 (Dec 2015 to May 2016)	Wave 5 (Oct 2016 to 31 Jan 2017)
Age	0/29	0/27	0/22	0/26	0/109
Sex	0/29	0/27	0/22	0/26	0/109
BMI	1/29	0/27	2/22	1/26	9/109
Poultry or live poultry market exposure	2/29	0/27	2/22	0/26	15/109
Severe infection	2/29	0/27	2/22	0/26	9/109
ICU admission	2/29	0/27	2/22	1/26	33/109
Death	0/29	2/27	2/22	1/26	0/109
Number of days from onset of disease to ICU admission	0/21	0/ 20	0/17	0/20	0/65
Number of days from onset of disease to death	0/10	0/13	0 /13	0/11	0/36

BMI: body mass index; ICU: intensive care unit.

## Results

### Human infections

**Figure 1:**
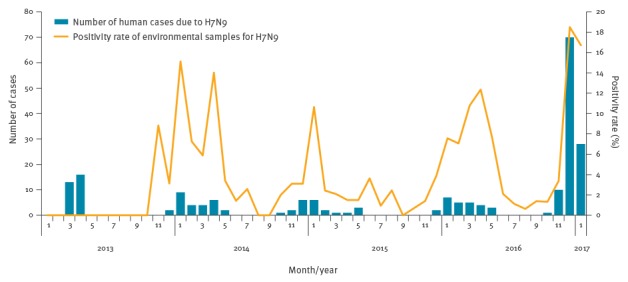
Number of human cases due to H7N9 and positivity rate of environmental samples for H7N9 in Jiangsu province, 2013–31 January 2017 (n = 213 cases)

Jiangsu province is now experiencing the fifth wave of human infections with H7N9. As of 31 January 2017, the cumulated number of cases in the current wave is 109 (since October 2016), which is overwhelmingly higher than in each of the previous four waves (29, 27, 22 and 26, respectively). The peak monthly incidence in the fifth epidemic is also higher than in the previous waves (70 vs 16, 9, 6 and 7) (Figure 1).

**Figure 2:**
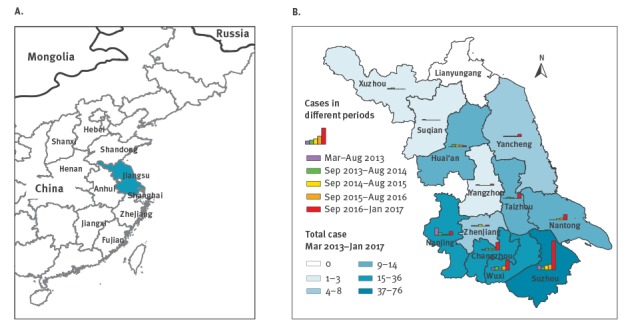
Location of Jiangsu province in eastern China, with neighbouring provinces (A) and geographical distribution of human infections with H7N9 in Jiangsu province (B), China, 2013–2017

Among recorded cases, 36 died. During the same period, a total of 305 patients were reported nationally, with 98 deaths. Patients of Jiangsu accounted for 35.7% (109/305) of those in the entire country, and this proportion was higher than in previous waves (8.9%–22.0%, p < 0.0001). The preliminary case fatality rate, as there were 36 patients still hospitalised at the time of this analysis, was 33.0% (36/109) in Jiangsu, which is slightly higher than that of the rest of China (31.6%, 62/196). Ten of 13 cities in Jiangsu province reported human infections during the fifth wave, which is also more than previously (7–9 cities). Most of the cases were reported from Suzhou (49/109, 45.0%), Wuxi (17/109, 15.6%) and Changzhou (13/109, 11.9%). These three cities are adjacent and all located in southern Jiangsu province (Figure 2).

In the fifth wave in Jiangsu province, male patients accounted for 74.3% (n=81) of the reported 109 patients, and the overall median age was 58 years. The proportion of severe infections as well as having poultry or poultry market exposure history remained high (93.0% and 70.8%, respectively) as in the previous waves. No significant differences were observed in patients’ demographic characteristics (age, sex and BMI), poultry or live poultry market exposure history, and proportion of severe infections and deaths between this wave and previous waves. However, patients’ disease progression seemed to be accelerating during late waves. The median time intervals from onset of disease to intensive care unit (ICU) admission were around 7 days during the latest three waves, which was shorter than that of the first and the second wave (10 and 9 days, p = 0.048). The median time interval from onset of disease to death was 13.5 days in the current wave, which was significantly shorter than that of previous four waves (15–28 days, p < 0.0001) (Table 3). 

**Table 3:** Comparisons of selected characteristics of H7N9 patients among five epidemic waves in Jiangsu province of eastern China, 2013–31 January 2017

Selected characteristics	Wave 1 (Mar to Apr 2013) n = 29	Wave 2 (Dec 2013 to May 2014) n = 27	Wave 3 (Oct 2014 to May 2015) n = 22	Wave 4 (Dec 2015 to May 2016) n = 26	Wave 5 (Oct 2016 to 31 Jan 2017) n = 109	P^a^
Age, years, median, (IQRs)	54 (35–70)	53 (42–66)	57 (50–68)	53 (41–63)	58 (46–66)	0.482
Proportion of male cases	21/29	20/27	14/22	18/26	81/109	0.860
BMI, median (IQRs)	22.94 (22.17–24.16)	23.44 (21.97–26.12)	23.85 (22.49–26.55)	24.01 (21.23–26.37)	24.22 (22.49–26.12)	0.472
Proportion of cases with poultry or live poultry market exposure	14/20	20/27	15/20	20/26	63/89	0.979
Proportion of severe infections	23/28	26/27	18/20	24/26	93/100	0.398
Proportions of ICU admission	21/27	20/27	17/20	20/25	65/76	0.665
Proportion of deaths	10/29	13/27	13/22	10/26	36/109^b^	0.586
Median number of days (IQR) from onset of disease to ICU admission	10 (7–14)	9 (8–11)	7 (6–9)	7.5 (6–10)	7 (6–10)	0.048
Medium number of days (IQR) from onset of disease to death	28 (20–45)	24 (20–38)	15 (13–23)	22.5 (13–42)	13.5 (8–20.5)	< 0.0001

BMI: body mass index; IQRs: interquartile ranges.

^a^ Pearson chi-squared test was used for comparing proportions and Kruskal–Wallis H test was used for comparing medians.

^b^ There were 36 patients still in hospital at the time of the study.

Three clustered human infections with H7N9 virus have been reported in previous waves in Jiangsu province, one in the first wave [11] and another two in the fourth (data not shown). During the fifth wave, another cluster of probable human-to-human transmission with laboratory evidence (viral sequence similarity > 99.99%) was reported in Suzhou city. The index case, a person aged in their mid-60s, visited live poultry markets regularly before getting ill. Three days after this case’s onset of illness, a relative in their late 30s, took care of the index patient while in hospital, for a duration of three days, without taking any precautions. The relative had contact with sputum and the body of the index patient during this period and developed symptoms six days later. Two days after symptom onset, this secondary case was also admitted to hospital. Both patients were severely ill with diagnosis of pneumonia. The secondary patient recovered and was discharged from the hospital 25 days after their illness started, but the index patient died 17 days after disease onset.

Seasonal influenza activity peaked with H3N2 being the predominant subtype during the same period (December 2016) in Jiangsu province. Similar to 2013, when we reported a patient co-infected with H7N9 and H3N2 virus [12], another such patient was identified in 2016 using real-time PCR. The patient, aged in their late 50s lived in Suzhou, without chronic medical conditions and without seasonal influenza vaccination history. Three days prior to symptom onset, this person had visited a live poultry market together with their spouse and bought two chickens and one goose, without direct contact with these live poultry. Seven days after disease onset, the patient was admitted to hospital with severe pneumonia and died 13 days later. The spouse did not develop any symptoms.

### Environmental virus detection

The environmental H7N9 virus detection rate was found significantly elevated in Jiangsu during this wave as well (p < 0.0001). It peaked at 18.5% (149/805) in December 2016 when most of the human infections of the ongoing fifth wave were reported, while the rate was only 3.1% (2/65), 3.1% (11/359) and 3.9% (22/561) during the same period of 2013, 2014 and 2015, respectively (Figure 1). In addition, an increased detection of H7N9 virus in environmental samples collected during the summer was noted. The virus was not detected in August and September in 2014 and in September in 2015, but was detected in both August and September in 2016 (Figure 1).

### Meteorological impact

We found that there were nine days with high risk minimum temperature (provincial mean) in December 2016 when most of the human infections were reported, which was significantly more than that of the same period in past three years (0, 0 and 4 in 2013, 2014 and 2015 respectively, p < 0.0001). The number of days with high risk maximum temperature (provincial mean) in December 2016 was also significantly more than in the past 3 years (10 vs 5, 2 and 3 days respectively, p = 0.028). In November 2016 however, while the numbers of ‘high risk’ days were similar to that of December 2016 (10 days vs 9 days for minimum temperature, 12 days vs 10 days for maximum temperature), the environmental H7N9 virus detection rate was remarkably lower than that of December (3.38% vs 18.50%), as was also the rate of human infections (9 in November vs 70 in December). The daily temperature data of January 2017 are unavailable at present. 

### Viral analyses

**Figure 3:**
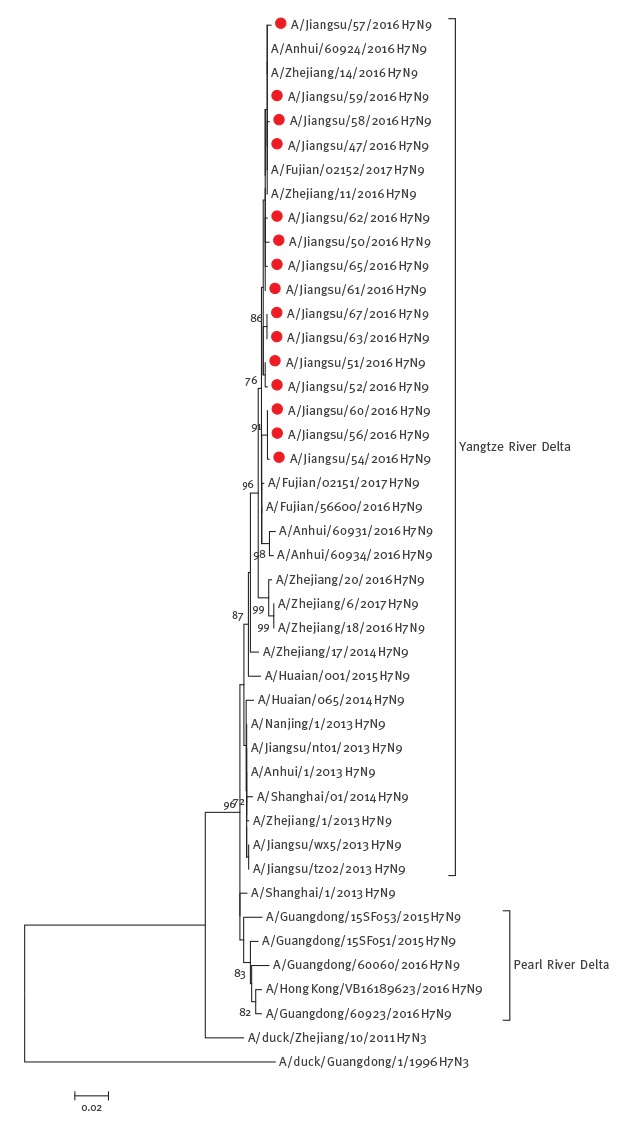
Phylogenetic analysis of haemagglutinin (HA) sequences of H7N9 viruses derived from patients reported from October to December 2016 in Jiangsu province and H7N9 and H7N3 HA sequences identified earlier in China, 1996–2016

Whole genomes of fifteen H7N9 strains, which were isolated from patients reported during this wave (1 in October, 2 in November and 12 in December 2016) in Jiangsu province, were sequenced and analysed. All the strains were from severely affected cities, that is, Suzhou (n = 10 total cases), Changzhou (n = 3) and Wuxi (n = 2). A phylogenetic tree of the HA genes of these strains was produced together with viruses isolated previously. The results showed that viruses isolated during the current wave shared the same ancestor as earlier viruses from 2013 to 2015, but clustered in an independent clade (Figure 3), which suggested that H7N9 virus is continuously evolving.

The phylogenetic tree of the HA1 coding nt sequences was generated by Molecular Evolutionary Genetic Analysis (MEGA) version 6.06 by neighbour-joining method with 1,000 bootstrap replicates; only bootstrap values over 70 are shown. Red circles indicate H7N9 viruses isolated from Jiangsu province, 2016.

Genetic characteristic analysis indicated that significant mutations have not occurred in these H7N9 viruses. No substitutions were observed in two positions (G186V and Q226L) located in the receptor-binding sites of HA, indicating that the virus retains the ability to bind with both avian α2,3-sialic acid and human α2,6-sialic acid receptors. The R294K mutation in the neuraminidase (NA) protein, which is believed to confer resistance to NA inhibitors [13] was not observed in these isolates. Furthermore, the substitution of E627K in polymerase basic protein 2 (PB2) protein was not observed either, which indicates that viruses could not efficiently replicate in human [14].

## Discussion

Jiangsu province is now experiencing the fifth wave of human infections with H7N9, with a significantly elevated number of cases. Visiting live poultry markets is the main risk factor for H7N9 infection for the public, due to poultry contact in this setting or environmental contamination [15,16]. The high H7N9 virus detection rate in these sites may directly contribute to the elevated human H7N9 infections. Therefore, live poultry market closures have been suggested as an effective method to control such infections [17,18]. Accordingly, several municipal governments in Jiangsu province, including Suzhou, Wuxi and Changzhou, have implemented temporary comprehensive live poultry market closures since December 2016. Subsequently, a significant decline in human infections has been observed in these cities/municipalities, as well as province-wide since January 2017 (Figure 1). The decrease was even more evident in February (data not shown).

Many meteorological factors, such as temperature, relative humidity [7,19], specific humidity [20] and solar radiation [21], have been reported to influence influenza activity. As for H7N9, both daily minimum and daily maximum temperatures have been reported to contribute significantly to human infection, but not relative humidity [22]. Other meteorological factors have not been reported. The overall impact of ambient temperature on human infection rates with H7N9 may nevertheless also depend on the underlying level of environmental H7N9 virus contamination, as exemplified by the results in November 2016, when although temperatures appeared to be permissive to human infection, low rates were observed, coinciding with low rates of environmental contamination. The environmental H7N9 contamination rate could be influenced by multiple factors, such as H7N9 virus infection rate of poultry for sale, and the hygiene level of the live poultry market. The interaction and correlation between temperature and other factors and their impact on human infections need to be investigated further in future studies.

Antivirals such as oseltamivir were administrated to almost all of the H7N9 patients in recent years in Jiangsu province. Furthermore, the time interval from onset of disease to antiviral administration is becoming shorter due to promoted sensitivity of clinicians. Clinicians also gained experiences in treatment, such as the rational use of ventilators and extracorporeal membrane oxygenation (ECMO). All of these measures are beneficial for the patients’ clinical outcome. Nevertheless, an accelerated disease progression of H7N9 patients during latest waves was still observed, which suggests that the viral pathogenicity might have become stronger. In addition, the increased detection rate of H7N9 in environmental samples suggests that the virus might become more resistant to high ambient temperature. Although no significant mutations were observed in key functional loci of the isolates from the current wave in our preliminary analyses, further work still needs to be conducted in detail. For instance, changes in the length of the neuraminidase stalk region might impact virulence [23] and residues 41V and/or 210D in the nucleoprotein (NP) protein could enhance polymerase activities and potential replication at low temperature [24].

The pandemic potential of the H7N9 virus needs to be closely watched. In humans, co-infection of this virus with seasonal influenza might provide reassortment opportunities for the emergence of a new pandemic virus. In addition, the continuous mutation and reassortment of H7N9 with other avian influenza viruses lately resulted in the identification of H7N9 isolates with characteristics of high pathogenicity to poultry, which was concerning for the poultry industry [25]. There is also a risk that H7N9 might acquire better ability of spreading from poultry to ducks and wild birds, and thus be disseminated worldwide, threatening humans in a much wider geographical range [26-28]. Therefore, it is critical to control the transmission of H7N9 virus in poultry to lower these risks.

To avoid the possibility of further adaption to human of this virus, early identification of human infections with H7N9 and early administration of neuraminidase inhibitors are critically needed. At present, the median time intervals from onset of disease to first medical consultation and from onset of disease to administration of neuraminidase inhibitors are two and six days, respectively. Efforts implementing effective rapid diagnostic kits in primary medical facilities, such as community clinics, could further promote the timeliness of diagnosis and antiviral therapy, as nearly half of the H7N9 patients first seek medical services in these facilities.

Until now, older males still account for most of the H7N9 patients. An overwhelming majority of the reported patients were severely infected and the overall case fatality remained above 30%. Live bird markets are the most common sites for the public to contact birds or bird materials which might carry H7N9 virus. With the continuous closures of live bird markets, the case number is expected to keep decreasing. In addition, the upcoming warmer weather would also deter the transmission of H7N9. However, we should be alert that H7N9 cases might occur in areas where live bird market closures are not implemented, also because live poultry from places affected by H7N9 and with market closures, may be transferred to these areas. A full investigation of the current wave of human infections with H7N9 virus is still ongoing. This study presents timely preliminary results, including possible causes, which could help researchers in further detailed analyses.
